# Massive Renal Cyst Presenting With Chest Pain and Gastrointestinal Symptoms

**DOI:** 10.7759/cureus.89442

**Published:** 2025-08-05

**Authors:** Sean Twardy, Seti Zepeda, Harjinder Kumar

**Affiliations:** 1 School of Medicine, Uniformed Services University of the Health Sciences, Bethesda, USA; 2 Internal Medicine, Walter Reed National Military Medical Center, Bethesda, USA

**Keywords:** atypical presentation, benign cystic lesions, gastro-intestinal symptoms, intra-abdominal mass, massive renal cyst, percutaneous aspiration, percutaneous drainage, renal cyst, simple renal cyst

## Abstract

Renal cysts are common, typically asymptomatic, fluid-filled sacs that rarely require intervention. Nevertheless, in rare cases, large symptomatic cysts can cause significant morbidity. We report the case of an 87-year-old man presenting to the emergency department with right chest wall pain following a ground-level fall, accompanied by worsening nausea, vomiting, and decreased oral intake over 6-7 months. A computed tomography scan revealed a massive right-sided renal cyst (18.6 cm x 11.5 cm x 6 cm) causing displacement of abdominal contents through mass effect. Following consultation with Urology and Interventional Radiology, percutaneous aspiration was performed, resulting in symptom resolution. This case underscores the rare presentation of a massive renal cyst with chest pain and gastrointestinal symptoms, highlighting the need to consider this diagnosis in similar clinical scenarios and the efficacy of percutaneous aspiration as a minimally invasive treatment.

## Introduction

Renal cysts are typically asymptomatic and are commonly discovered incidentally during imaging. They are frequently described using the Bosniak classification system in CT and MRI studies. Ultrasound studies have shown that renal cysts have a prevalence of approximately 10.7% in the population, increasing to over 35% in those aged 70 and older [1]. Similarly, in a CT study for presentations unrelated to renal or liver investigations, the prevalence of incidentally discovered simple renal cysts has been reported to be as high as 41% [2]. While most require no intervention, large or symptomatic cysts can cause significant morbidity, including pain or mass effect on adjacent structures [3]. Massive renal cysts exceeding 15 cm are rare and may present with atypical symptoms, complicating diagnosis. This case report describes an 87-year-old man with a massive 18.6 cm renal cyst causing right-sided chest pain worsened by inspiration and gastrointestinal symptoms due to mass effect, a presentation unreported in the literature to our knowledge. The case highlights diagnostic challenges and the efficacy of percutaneous aspiration as a minimally invasive treatment.

## Case presentation

An 87-year-old man presented to the emergency department with persistent right-sided chest wall pain (8/10 severity, worsened by inspiration) one week after a ground-level fall onto his right side while unloading groceries. He reported nausea, vomiting, decreased oral intake, and voiding difficulties over the past 6-7 months, describing a sensation of food lodging in his lower throat, leading to intermittent vomiting and reliance on laxatives for bowel movements. His medical history included hypertension, benign prostatic hyperplasia, chronic kidney disease, and laryngeal carcinoma (status post esophagectomy with gastric pull-through and radiation therapy in 2005). Medications included cyanocobalamin, ergocalciferol, felodipine, finasteride, losartan, and tamsulosin. Vital signs were stable: temperature 36.7°C, heart rate 62 beats/min, respiratory rate 16 breaths/min, blood pressure 126/69 mmHg, oxygen saturation 97%, height 162 cm, and weight 45.4 kg. Physical examination revealed tenderness on the right lateral chest wall; other findings were unremarkable.

The differential diagnosis included musculoskeletal injury (e.g., rib fracture), pleuritic pain, myocardial infarction, pulmonary embolism, malignancy recurrence, or intra-abdominal pathology, given his chronic gastrointestinal symptoms and cancer history. The diagnostic workup comprised an ECG, comprehensive metabolic panel (CMP) with estimated glomerular filtration rate (eGFR), lipase, troponin-T, prothrombin time/international normalized ratio (PT/INR), partial thromboplastin time (PTT), magnesium, urinalysis with microscopy, and a non-contrast CT scan of the chest, abdomen, and pelvis. Laboratory results showed normocytic anemia, leukocytosis (elevated WBC and neutrophil percentage), impaired renal function (elevated blood urea nitrogen (BUN), creatinine; low eGFR), low calcium and albumin, and elevated troponin-T, possibly due to reduced renal clearance. Urinalysis suggested a urinary tract infection. Compared to a prior encounter with simple cystitis, BUN levels had increased (Table 1). An esophagogastroduodenoscopy (EGD) was performed, revealing multi-level strictures and fibrotic changes in the proximal esophagus secondary to past radiation treatment. Dilation of the proximal esophageal strictures was not performed. Additionally, a sliding hiatal hernia with pooled secretions and dissolving pill contents was noted.

**Table 1:** Patient laboratory values on admission (July 24th, 2024) compared with those from a previous encounter during which the patient was diagnosed and treated for a urinary tract infection. MCV: mean corpuscular volume; MCH: mean corpuscular hemoglobin; MCHC: mean corpuscular hemoglobin concentration; RDW: red blood cell distribution width; MPV: mean platelet volume; PT: prothrombin time; PTT: partial thromboplastin time; INR: international normalized ratio; AGAP: anion gap; BUN: blood urea nitrogen; eGFR: estimated glomerular filtration rate; CKD-EPI: Chronic Kidney Disease Epidemiology Collaboration; UA: urinalysis.

Test	Reference range	Value on admission on July 24, 2024	Interpretation	Previous values prior to presentation	Interpretation
WBC	3.6-10.6	11	High	5.4	Normal
RBC	4.21-5.92	3.2	Low	3.31	Low
Hemoglobin	12.8-17.7	9.4	Low	9.6	Low
Hematocrit	37.5-50.9	27.9	Low	29	Low
MCV	79.5-96.8	87.2	Normal	87.7	Normal
MCH	26.2-33.1	29.5	Normal	29	Normal
MCHC	32.6-35.0	33.8	Normal	33.1	Normal
RDW	12.0-16.2	14.8	Normal	15.1	Normal
Platelets	162-427	280	Normal	188	Normal
MPV	7.0-10.9	7.1	Low	6.9	Low
Neutrophil%	40.7-76.4	79.5	High	62.5	Normal
Lymphocyte%	15.9-47.8	18.2	Normal	30.2	Normal
Monocyte%	4.5-11.8	1.7	Low	5.2	Normal
Eosinophil%	0-4.0	0.1	Normal	1.3	Normal
Basophil%	0-4.0	0.5	Normal	0.5	Normal
Neutro absolute	1.8-7.5	8.8	High	3.8	Normal
Lymph absolute	1.0-3.1	2	Normal	1.8	Normal
Mono absolute	0.2-0.8	0.2	Normal	0.3	Normal
Eos absolute	0-0.5	0	Normal	0.1	Normal
Baso absolute	0-0.4	0.1	Normal	0	Normal
PT	9.8-11.4	11.9	High	-	-
PTT	22.5-31.5	29.6	Normal	-	-
INR		1.1	Normal	-	-
Sodium	136-145	139	Normal	139	Normal
Potassium	3.5-5.1	4.7	Normal	4.4	Normal
Chloride	98-107	105	Normal	106	Normal
CO_2_	22.0-29.0	21	Low	20	Low
AGAP	7.0-16.0	18	High	13	Normal
BUN	7.0-20.0	45.2	High	20.8	High
Creatinine	0.7-1.2	1.96	High	1.85	High
eGFR CKD-EPI		32	Low	35	Low
Glucose	74-109	100	Normal	97	Normal
Calcium	8.8-10.2	8.7	Low	9.3	Normal
Protein total	6.6-8.7	8.1	Normal	-	-
Albumin	3.5-5.2	3.2	Low	3.6	Normal
Magnesium	1.6-2.4	2.3	Normal	-	-
Bilirubin total	0-1.2	0.69	Normal	-	-
Lipase	13-60	17	Normal	-	-
Alk Phos	40-129	78	Normal	-	-
ALT	0-50	<5	Low	-	-
AST	0-50	Not Done	-	-	-
Troponin-T	0-19	27.55	High	-	-
UA color	Yellow	Yellow	Normal	Yellow	Normal
UA clarity	Clear	Hazy	Abnormal	Cloudy	Abnormal
UA pH	5.0-8.0	5	Normal	6	Normal
UA spec gravity	1.001-1.026	1.012	Normal	1.01	Normal
UA glucose	Negative	Negative	Normal	Negative	Normal
UA ketones	Negative	Negative	Normal	Negative	Normal
UA blood	Negative	Small	Abnormal	Small	Abnormal
UA protein	Negative	100	Abnormal	100	Abnormal
UA bili	Negative	Negative	Normal	Negative	Normal
UA urobilinogen	0-0.2	2	Abnormal	0.2	Normal
UA nitrite	Negative	Negative	Normal	Negative	Normal
UA leuk esterase	Negative	Moderate	Abnormal	Moderate	Abnormal
UA WBC	0-5	>40	Abnormal	69	High
UA RBC	0-4	0-2	Normal	2	Normal
UA bacteria	None	Moderate	Abnormal	Many	Abnormal
UA Epi Squam	0-5	0-2	Normal	0	Normal

**Figure 1:**
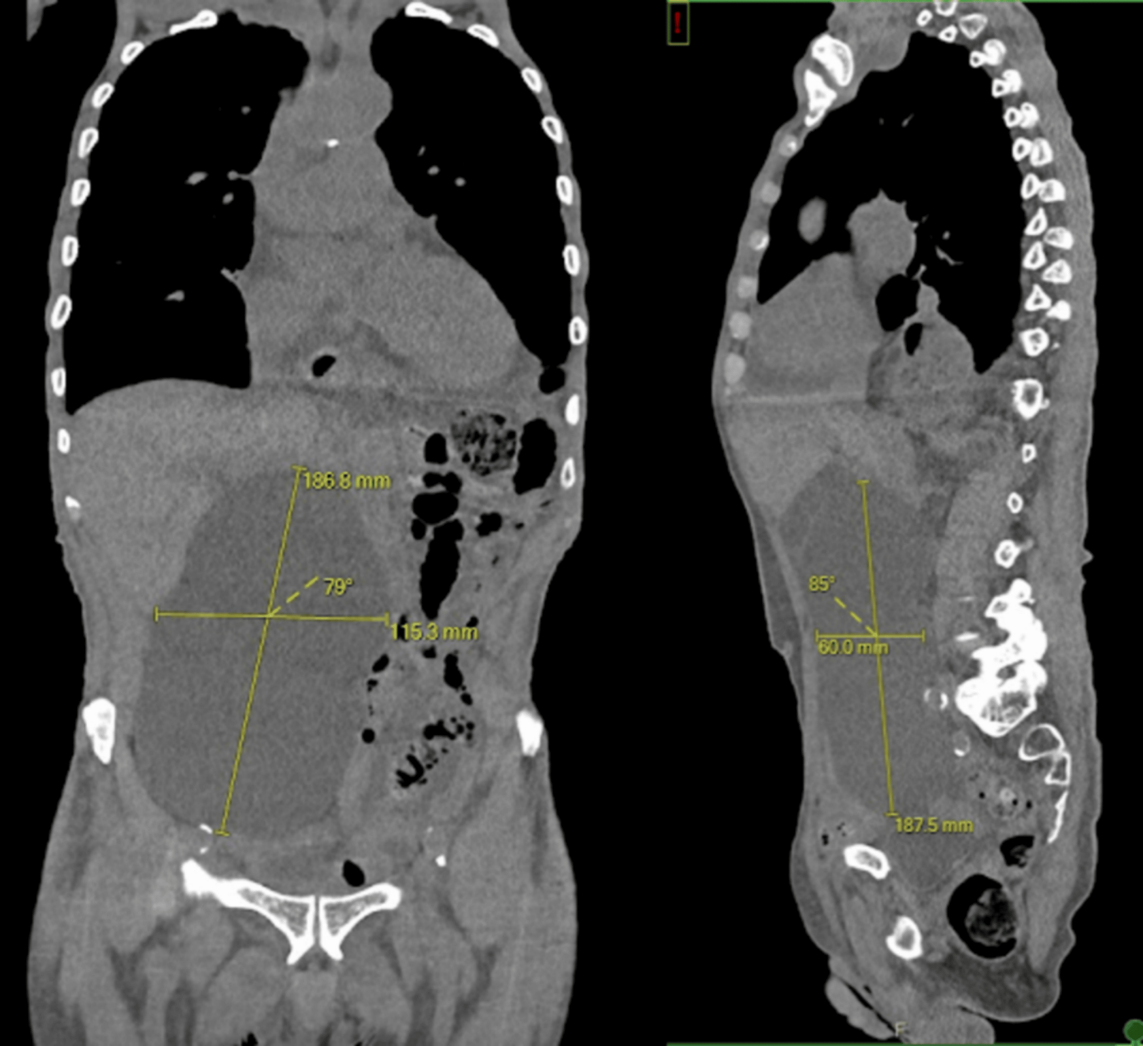
Computed tomography (without contrast) of the abdomen showing the patient's massive renal cyst in coronal and sagittal views. In the largest dimensions, the cyst measures 18.6 cm x 11.5 cm x 6 cm.

The CT scan of the chest, abdomen, and pelvis revealed multiple, bilateral simple renal cysts. The largest was a right-sided renal cyst without complex features, measuring 18.6 cm x 11.5 cm x 6 cm. It extended from the lower pole and had a mass effect on the right peritoneal cavity, displacing the contents towards the left. There was no hydroureteronephrosis present, and there was no evidence of a small bowel obstruction given the lack of dilated loops of small bowel, transition point, or air-fluid levels (Figure 1).

**Figure 2:**
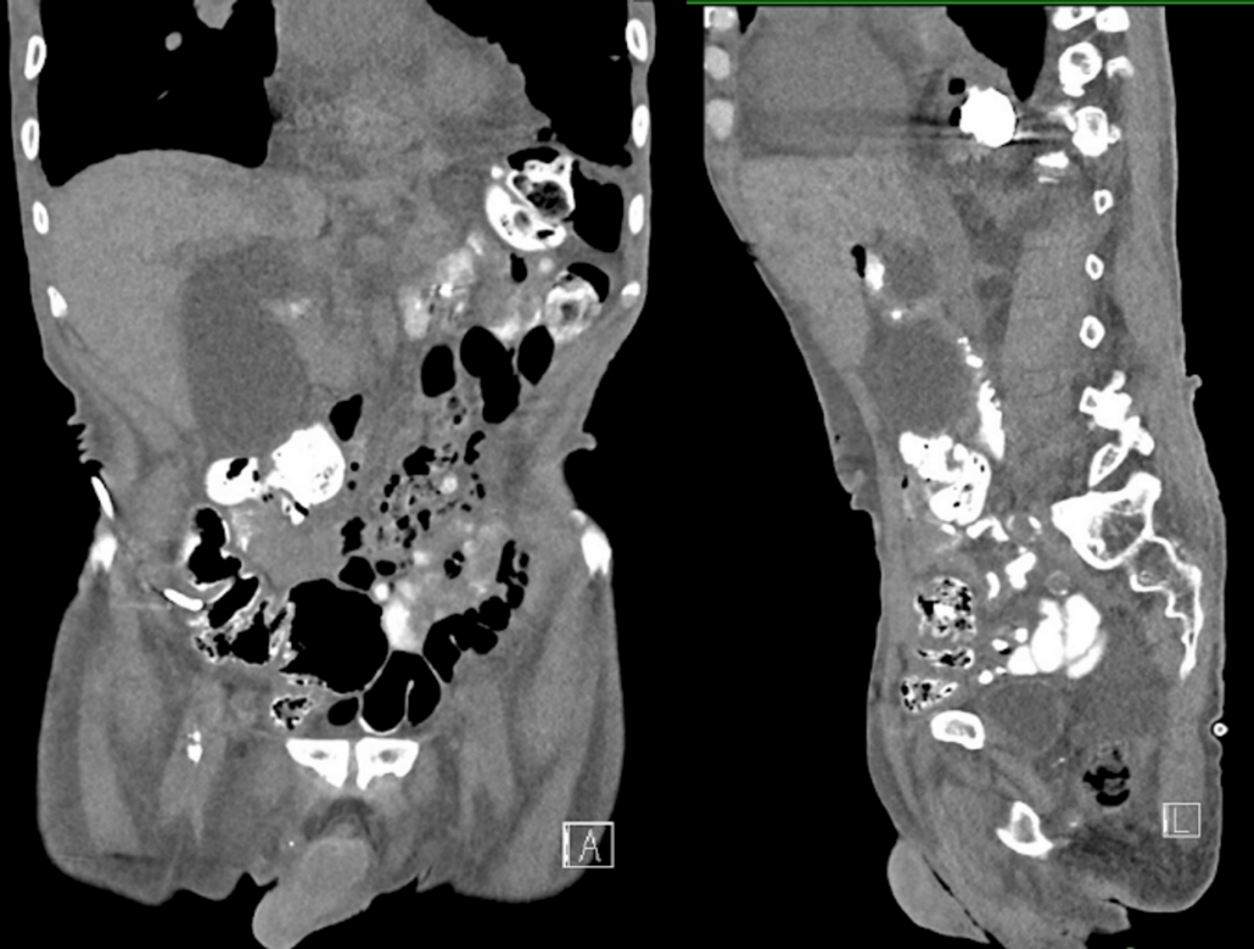
Computed tomography (without contrast) of the abdomen following drainage of the patient’s right renal cyst with coronal and sagittal views.

After consultation with Urology and Interventional Radiology, the patient consented to percutaneous aspiration of the massive renal cyst. Under local anesthesia, an 8.5 French drainage catheter was placed into the cyst, draining 500 mL of amber fluid. Two loculations were separately aspirated using a 5 French Yueh needle, yielding 350 mL and 150 mL of serous fluid, respectively, for a total of 1000 mL. Post-procedure imaging confirmed near-complete drainage (Figure 2). During hospitalization, the patient’s chest pain, inspiratory discomfort, nausea, and vomiting resolved, with repeat CT showing significant reduction in cyst size.

## Discussion

Renal cysts are common, typically asymptomatic, and often discovered incidentally during imaging studies [1]. However, when cysts become large, as in our case of an 18.6 cm lesion, they can exert significant mass effect, resulting in symptoms that mimic other abdominal or thoracic pathologies [3]. The presentation of right-sided chest pain in our patient likely resulted from liver and diaphragmatic irritation or displacement due to the superior expansion of the cyst, a phenomenon that is rare, but plausible given prior reports of compressive effects from large cysts [3,4]. We suggest that the pleuritic chest pain in our patient may have resulted from downward movement of the diaphragm and liver against the cyst during inspiration. In a report on a 77-year-old woman with a massive renal cyst, Mohammed et al. reported that the presenting symptom for their patient was right flank pain [3]. The authors further summarize that in previous case reports the presenting symptoms from these cysts have been varied, with documented instances of abdominal pain, suprapubic pain, and large bowel obstruction.

Gastrointestinal symptoms such as nausea, vomiting, and reduced oral intake may potentially be attributable to bowel compression, as described in prior cases of massive renal cysts with unusual presentations [3,4]. Massive renal cysts can cause significant displacement of intra‑abdominal organs, including the bowel, which may contribute to gastrointestinal symptoms in affected patients [3]. Additionally, at 45.4 kg, the patient's smaller body habitus may have increased his susceptibility to compressive symptoms from the large renal cyst. It is possible that some of his enteral symptoms were related to his previous history of esophageal cancer and fibrotic changes discovered on EGD; nevertheless, his treatment for esophageal cancer was completed in 2005, and the remote history makes this less likely.

Hypertension, a common symptom of massive renal cysts in previous reports [3], was absent in our patient. Through mass effect, cyst compression of renal vasculature or parenchyma can reduce renal perfusion, leading to a resultant activation of the renin-angiotensin-aldosterone system (RAAS) [5]. In a cross-sectional study by Lee et al., subjects with simple renal cysts had a higher risk of prehypertension and hypertension independent of cardiovascular and metabolic risk factors. Furthermore, subjects with two or more cysts and a cyst size of at least 2 cm were at greater risk than those with just one simple renal cyst [5]. With greater cyst burden or volume, patients with massive renal cysts are likely at significantly higher risk for prehypertension or hypertension. 

Management options for symptomatic renal cysts include percutaneous aspiration, with or without sclerotherapy, or surgery [5,6]. Long-term observational data show that while most renal cysts grow slowly, a subset, particularly in younger patients or those with multiloculated cysts, such as our patient, may exhibit faster enlargement, supporting the role of intervention when symptoms or rapid growth occur [7]. Percutaneous aspiration, with or without sclerosants, is often a reasonable initial treatment; it is minimally invasive, safe, and cost-effective [5]. Kim et al. demonstrated that single-session ethanol sclerotherapy with a 40-minute retention technique is effective and low risk for treating simple renal cysts [8]. In general, percutaneous aspiration remains the most common approach for such cases due to its simplicity and efficacy [3]. 

Laparoscopic decortication is a surgical treatment option that has been associated with a reduced risk of overall cyst recurrence in comparison with percutaneous aspiration [9]. In a study by Bas et al. of 184 patients with symptomatic renal cysts, higher success rates were reported with laparoscopic decortication compared to aspiration and sclerotherapy [6]. In this study, however, few cysts were over 15 cm, limiting generalizability to massive cysts. Nevertheless, the risks of surgery must be considered in the context of the individual patient, with attention given to the patient’s comorbidities, ability to tolerate surgery, and individual preferences.

Percutaneous aspiration without sclerotherapy was chosen for our patient through a process of shared decision-making, prioritizing a minimally invasive approach that would lead to symptom resolution. In the event of recurrence, our patient could undergo repeat percutaneous aspiration or potentially opt for surgical management, such as laparoscopic decortication. Recurrence rates for simple renal cysts treated without sclerosants have been reported to be between 20% and 80% [10]. Raskin et al. reviewed the effectiveness of conservative and radiologic strategies and noted that percutaneous aspiration and sclerotherapy remain first-line treatments for symptomatic simple renal cysts, with surgery considered only when these approaches fail [11]. For our patient, we reiterate our support for the decision for initial percutaneous aspiration. In the instance of recurrence, we would likely favor repeat percutaneous aspiration via shared decision-making in the context of this patient’s age, comorbidities, and goals of care.

## Conclusions

This case describes an 87-year-old man with an 18.6 cm renal cyst causing right-sided chest pain worsened by inspiration and gastrointestinal symptoms due to mass effect, a rare presentation. It underscores the importance of considering massive renal cysts in patients with atypical chest or abdominal complaints. Percutaneous aspiration proved effective and minimally invasive, offering a viable treatment option. Long-term follow-up with imaging is needed to monitor for recurrence. For our patient, we recommended a repeat non-contrast CT scan in six months.

## References

[1] Chang CC, Kuo JY, Chan WL, Chen KK, Chang LS (2007). Prevalence and clinical characteristics of simple renal cyst. J Chin Med Assoc.

[2] Carrim ZI, Murchison JT (2003). The prevalence of simple renal and hepatic cysts detected by spiral computed tomography. Clinical Radiology.

[3] Mohammed KM, Zarm A, Velez JC, Mohamed MM (2023). Massive renal cyst displacing intra-abdominal structures. Ochsner J.

[4] Riyach O, Ahsaini M, Tazi K (2014). A huge renal cyst mimicking ascites: a case report. BMC Res Notes.

[5] Lee CT, Yang YC, Wu JS, Chang YF, Huang YH, Lu FH, Chang CJ (2013). Multiple and large simple renal cysts are associated with prehypertension and hypertension. Kidney Int.

[6] Bas O, Nalbant I, Can Sener N (2015). Management of renal cysts. JSLS.

[7] Terada N, Arai Y, Kinukawa N, Terai A (2008). The 10-year natural history of simple renal cysts. Urology.

[8] Kim JH, Jeon UB, Jang JY, Kim TU, Ryu H, Yeom JA, Roh J (2022). Efficacy of single-session 99.5% ethanol sclerotherapy for incidentally found simple renal cysts. Medicine (Baltimore).

[9] Kobayashi K, Yoneyama S, Iwasa E, Karibe J, Yamashita D, Takizawa A (2022). A case involving laparoscopic decortication of a large simple renal cyst using conventional monopolar device. Int J Surg Case Rep.

[10] Garfield K, Leslie SW (2025). Simple renal cyst. StatPearls [Internet].

[11] Raskin MM, Poole DO, Roen SA, Viamonte M Jr (1975). Percutaneous management of renal cysts: results of a four-year study. Radiology.

